# Diarrheal Pathogens Associated With Growth and Neurodevelopment

**DOI:** 10.1093/cid/ciaa1938

**Published:** 2021-01-05

**Authors:** Jeffrey R Donowitz, Jeannie Drew, Mami Taniuchi, James A Platts-Mills, Masud Alam, Tahsin Ferdous, Talat Shama, Md Ohedul Islam, Mamun Kabir, Uma Nayak, Rashidul Haque, William A Petri

**Affiliations:** 1 Division of Pediatric Infectious Diseases, Children’s Hospital of Richmond at Virginia Commonwealth University, Richmond, Virginia, USA; 2 Division of Infectious Diseases and International Health, University of Virginia, Charlottesville, Virginia, USA; 3 Division of Parasitology, International Centre for Diarrhoeal Disease Research, Bangladesh , Dhaka, Bangladesh; 4 Department of Public Health Sciences and Center for Public Health Genomics, University of Virginia, Charlottesville, Virginia, USA

**Keywords:** diarrhea, stunting, neurodevelopment, low-income countries

## Abstract

**Background:**

Diarrheal pathogens have been associated with linear growth deficits. The effect of diarrheal pathogens on growth is likely due to inflammation, which also adversely affects neurodevelopment. We hypothesized that diarrheagenic pathogens would be negatively associated with both growth and neurodevelopment.

**Methods:**

We conducted a longitudinal birth cohort study of 250 children with diarrheal surveillance and measured pathogen burden in diarrheal samples using quantitative polymerase chain reaction. Pathogen attributable fraction estimates of diarrhea over the first 2 years of life, corrected for socioeconomic variables, were used to predict both growth and scores on the Bayley-III Scales of Infant and Toddler Development.

**Results:**

One hundred eighty children were analyzed for growth and 162 for neurodevelopmental outcomes. Rotavirus, *Campylobacter*, and *Shigella* were the leading causes of diarrhea in year 1 while *Shigella*, *Campylobacter*, and heat-stable toxin–producing enterotoxigenic *Escherichia coli* were the leading causes in year 2. Norovirus was the only pathogen associated with length-for-age *z* score at 24 months and was positively associated (regression coefficient [RC], 0.42 [95% confidence interval {CI}, .04 to .80]). Norovirus (RC, 2.46 [95% CI, .05 to 4.87]) was also positively associated with cognitive scores while sapovirus (RC, –2.64 [95% CI, –4.80 to –.48]) and typical enteropathogenic *E. coli* (RC, –4.14 [95% CI, –8.02 to –.27]) were inversely associated. No pathogens were associated with language or motor scores. Significant maternal, socioeconomic, and perinatal predictors were identified for both growth and neurodevelopment.

**Conclusions:**

Maternal, prenatal, and socioeconomic factors were common predictors of growth and neurodevelopment. Only a limited number of diarrheal pathogens were associated with these outcomes.

## Teaser

Rotavirus, *Campylobacter*, and *Shigella* were the top causes of diarrhea in year 1 while *Shigella*, *Campylobacter*, and heat-stable toxin–producing enterotoxigenic *Escherichia coli* predominated in year 2. Norovirus was associated with improved growth and cognitive scores. Sapovirus and enteropathogenic *E. coli* were inversely associated with cognitive scores.

Diarrhea remains the second leading cause of death among children aged <5 years worldwide [1]. While the attributable mortality due to diarrhea has declined over the last 2 decades, diarrheal incidence has declined more slowly [2, 3]. The change in diarrhea-associated mortality is likely due to increased oral rehydration salt and zinc coverage in low- and middle-income countries (LMICs), but the infectious burden remains high [4, 5]. The nondiarrheal effects of enteric pathogens, including poor linear growth and neurodevelopment, remain largely unaffected [6–10].

Recent work has highlighted the interconnected nature of stunting and neurodevelopmental delay, their overlapping but distinct pathogeneses, and their effects across all age groups of childhood [11, 12]. Approximately 23.8% of the world’s children <5 years old are stunted, defined as length-for-age *z* score (LAZ) ≤ –2 standard deviations [13]. Stunting has a hazard ratio of 2.28 for death prior to the age of 5 years with severe stunting conferring a hazard ratio of 5.48 [14]. Stunting is associated with neurodevelopmental delays, which may explain the link between poor growth and low productivity [15–17].

The processes leading to delayed neurodevelopment may be distinct from those leading to poor growth, although overlap exists. Febrile illness and systemic inflammatory cytokines have been associated with lower neurodevelopmental scores in LMIC children [18]. Chronic inflammation has been associated with aberrant facial-recognition responses in Bangladeshi toddlers [19]. Diarrheal disease remains one of the largest drivers of inflammation in the pediatric LMIC setting and in several studies of LMIC children, it was directly associated with poor neurodevelopment [6, 8, 20].

Here we present findings from a cohort of infants followed from birth to 2 years of age in urban Bangladesh on the relationship between diarrheagenic pathogens and both growth and neurodevelopment. Our hypothesis was that certain etiologies of diarrhea would be associated with decreased growth and neurodevelopment.

## PARTICIPANTS AND METHODS

We conducted a prospective, observational, and longitudinal cohort study in an urban neighborhood of Dhaka, Bangladesh, which recruited from June 2014 until March 2016. The primary outcome of this study was to describe the effect of cryptosporidiosis on growth and development, which has been published elsewhere [21]. The study was conducted in the Mirpur neighborhood of Dhaka, Bangladesh. The majority of homes in Mirpur are brick with tin roofs. Open sewers flow throughout the neighborhood.

Field research assistants visited the homes of each participant twice weekly to conduct diarrheal surveillance. If caregivers reported a diarrheal illness, defined as >3 unformed stools in a 24-hour window and separated from a previous diarrheal episode by at least 3 days, stool was collected. Stools were kept in coolers and transported to our field clinic where they were stored at 4°C. Each day the samples were transported, maintaining the cold chain, to the Parasitology Laboratory at the International Centre for Diarrhoeal Disease Research, Bangladesh (icddr,b), where they were aliquoted and placed in –80°C for storage until total nucleic acid (TNA) extraction.

Stool was tested for pathogen carriage using a TaqMan Array Card platform that tested for 36 enteric pathogens [22]. Two hundred milligrams of stool underwent TNA extraction using a slightly modified protocol of the QIAamp DNA Fast Stool Mini Kit (Qiagen, Gaithersburg, Maryland) [23, 24]. TNA was stored at –80°C until testing. Quantification cycle cutoff thresholds of <35 were used to indicate positivity [25]. A positive result was only considered valid if the corresponding extraction blank for that target was negative. A negative result was only considered valid if the positive controls were positive for the given sample. Each sample was spiked with phocine herpes virus (Erasmus Medical Center, Department of Virology, Rotterdam, the Netherlands) and bacteriophage MS2 (ATCC 15597B; American Type Culture Collection, Manassas, Virginia) as extrinsic controls for extraction and amplification.

*Escherichia coli* pathotypes were defined based on known virulence genes. Enteroaggregative *E. coli* (EAEC) was defined as aaiC and/or aatA, typical enteropathogenic *E. coli* (EPEC) as bfpA with or without eae, heat-stable toxin–producing enterotoxigenic *E. coli* (ST-ETEC) as STp and/or STh with or without LT, heat-labile toxin–producing enterotoxigenic *E. coli* (LT-ETEC) as LT only, Shiga toxin–producing *E. coli* (STEC) as stx1 and/or stx2 with or without eae, and *Shigella* as ipaH. Of note, ipaH can also define enteroinvasive *E. coli* (EIEC), but in previous work delineating *Shigella* from EIEC in Bangladesh, almost no EIEC was found, so in this analysis we assumed ipaH-positive samples to be *Shigella*.

Anthropometry was measured at study visits in our clinic every 3 months utilizing measuring boards and calibrated scales. Maternal anthropometry was measured using calibrated scales and stadiometers. Data on covariates including socioeconomic data, household data, and pregnancy data were collected via questionnaire at enrollment. Estimated gestational age was based on the Ballard neuromuscular maturity score, which was administered by staff trained in the procedure. Systemic and fecal biomarkers of inflammation were also included as covariates. C-reactive protein (CRP) was assessed in blood collected at 18 weeks via commercially available enzyme-linked immunosorbent assay (ELISA) kits (ALPCO, Salem, New Hampshire). Myeloperoxidase (ALPCO) and regenerating family member 1β (TechLab, Blacksburg, Virginia) were measured at week 22 of life in stool by commercially available ELISA. Soluble CD14 (sCD14) was measured in blood at week 18 using ELISA (R&D Systems, Minneapolis, Minnesota). LAZ and weight-for-age *z* score were calculated using the World Health Organization software WHO Anthro (version 3.2.2).

Neurodevelopmental assessment was made by a trained psychologist using a culturally adapted version of the Bayley-III Scales of Infant and Toddler Development. This tool assesses cognitive, motor (fine and gross), and language (receptive and expressive) development. Composite scores were used as outcomes in our analyses. This version of the Bayley-III was not normalized to the entire Bangladeshi population. Our group has utilized this tool in previous studies and it has been determined to have high short-term (within 7 days) retest reliability (*r* > 0.80) and high interobserver reliability (*r* = 0.99) [18, 26].

### Statistical Analysis

To calculate pathogen-specific burdens of diarrhea in the cohort, we calculated an adjusted attributable fraction (AF) of diarrhea for each pathogen. Specifically, we used a model developed for the Bangladesh site in the Global Enteric Multicenter Study (GEMS) to derive quantity-specific odds ratios for the association between each pathogen and diarrhea [27]. Using quantitative polymerase chain reaction data from 877 cases of moderate-to-severe acute diarrhea and age-, sex-, and village-matched controls in GEMS, we fitted a multivariable conditional logistic regression model to describe the association between pathogen quantity (using linear and quadratic terms) and diarrhea while adjusting for the presence of other pathogens. We then calculated AFs by summing the pathogen attributable fraction for each episode (AFe) across each of *j* cases in the current study—that is, ∑j1(1/j × AFei), where AFei = 1 – 1/ORi, and ORi is the quantity-specific odds ratio derived from the regression model. To estimate the variance for the model-based attribution, the odds ratios were estimated 1000 times using random perturbations of the model coefficients in accordance with their sampling variance-covariance. We derived 95% confidence intervals (CIs) from the 2.5th and 97.5th quantiles of the AF distribution, and the point estimate of the AF was calculated using the original model coefficients. The point estimate was also used to calculate the AFe for each individual episode of diarrhea.

We created linear regression models with LAZ at 1 and 2 years or Bayley scores as the outcomes. Separate models were created for each outcome but utilized the same predictors and covariates. Only pathogens with at least a 5% prevalence across all samples were included in the analysis. The only exception to this was *Cryptosporidium* spp, which was below the 5% prevalence cutoff in the first year of life but was kept due to the literature supporting this pathogen’s effect on growth [21, 28]. Initially models were run without selection. We then ran the models with a stepwise selection and forced all pathogen AFes into the final models as these were the primary predictors of interest. Only children with complete data sets for outcomes and covariates were included in the analyses (Supplementary Figure 1). R studio (version 1.1.456, packages dplyr and boot) was used for this analysis.

### Ethics Statement

This study was approved by the Research Review and Ethics Review Committees at the icddr,b. Informed consent was obtained from parents for their child’s participation in this study.

## RESULTS

### Pathogens

Two hundred fifty children were enrolled in this study. Two hundred twenty-eight children were followed through the first year of life with 814 diarrheal episodes recorded (mean, 3.6 episodes per child), of which 739 (90.8%) had pathogen analysis and AFes calculated. Two hundred ten children were followed through the second year of life. There were 659 episodes of diarrhea during the second year (mean, 2.2 episodes per child), of which 464 (67.8%) had pathogen analysis and AFe calculation. An average of 4.1 pathogens was detected per diarrheal stool sample.

**Figure 1.**
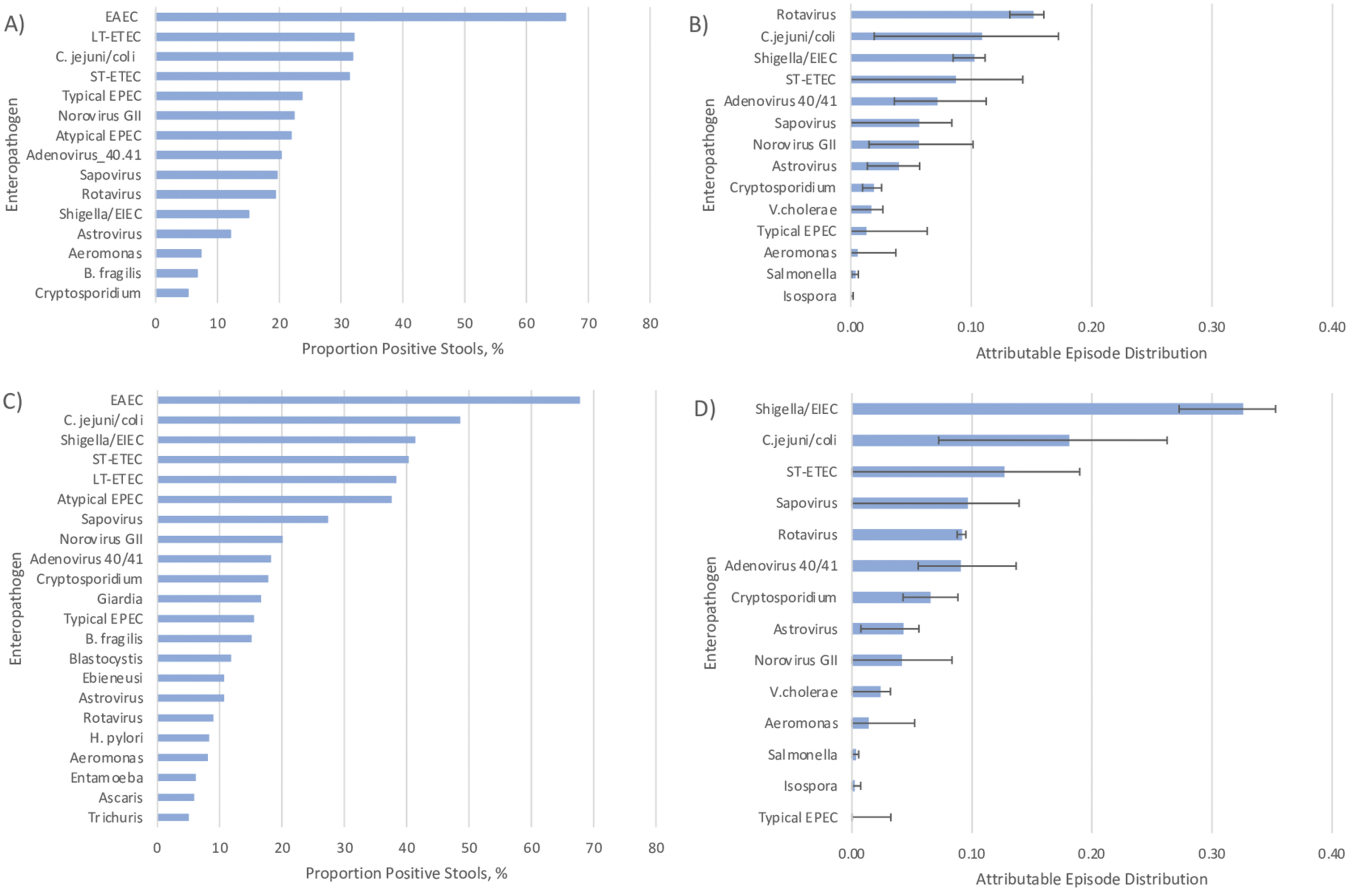
Incidence and number of pathogen attributable fraction estimates (AFes) for 0–12 months (*A* and *B*) and 13–24 months (*C* and *D*). The microbiologic etiology of diarrhea was determined by TaqMan Array Card polymerase chain reaction over the first 2 years of life. *A*, Proportion of stool samples positive for a given pathogen from birth to 12 months. *B*, Attributable episode distribution, plotted as median and 95% confidence interval, for a given enteropathogen from birth to 12 months. *C*, Proportion of stools positive for 13–24 months. *D*, Attributable episode distribution for 13–24 months. Enteroaggregative *Escherichia coli* (EAEC) was the most common pathogen detected in stool in the first 12 months of life followed by heat-labile toxin–producing enterotoxigenic *E. coli* (ETEC), and *Campylobacter jejuni/coli*. However, the most common causes of diarrhea were rotavirus followed by *C. jejuni/coli* and *Shigella*. EAEC remained the leading pathogen detected in the second year of life followed by *C. jejuni/coli* and *Shigella*. *Shigella* was the leading cause of diarrhea in the second year of life, followed by *C. jejuni/coli*, heat-stable toxin–producing ETEC, and sapovirus. Abbreviations: EAEC, enteroaggregative *Escherichia coli*; EPEC, enteropathogenic *Escherichia coli*; LT-ETEC, heat-labile toxin–producing enterotoxigenic *Escherichia coli*; ST-ETEC, heat-stable toxin–producing enterotoxigenic *Escherichia coli*; V. cholerae, *Vibirio cholerae*.

**Figure 2.**
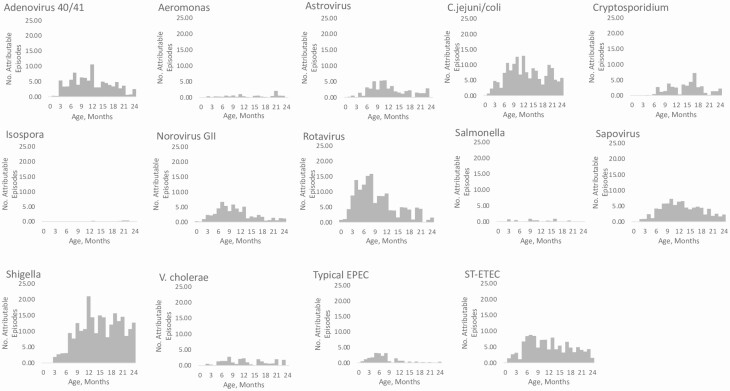
Number of attributable episodes by month of life. Attributable fraction estimates per month of life are shown for the pathogens analyzed. Bacterial pathogens tended to remain consistent throughout the first 2 years whereas viral pathogens tended to decrease in the second year of life. Abbreviations: C. jejuni/coli, *Campylobacter jejuni/coli*; EPEC, enteropathogenic *Escherichia coli*; ST-ETEC, heat-stable toxin–producing enterotoxigenic *Escherichia coli*; V. cholerae, *Vibirio cholerae*.

In year 1 EAEC was the most common pathogen detected in diarrheal stools (66.3%). EAEC was followed by LT-ETEC (32.1%), *Campylobacter jejuni/coli* (31.9%), ST-ETEC (31.2%), and typical EPEC (23.6%). Rotavirus had the largest AFe proportion followed by *C. jejuni/coli*, *Shigella*, ST-ETEC, and adenovirus 40/41. In the second year of life, EAEC remained the most detected pathogen, present in 67.8% of diarrheal samples, followed by *C. jejuni/coli* (48.6%), *Shigella* (41.4%), ST-ETEC (40.3%), and LT-ETEC (38.3%). *Shigella* spp had the highest AFe proportion followed by *C. jejuni/coli*, ST-ETEC, sapovirus, and rotavirus (Figure 1). Bacterial infections were relatively uncommon in the first 2 months of life but tended to increase from 2 to 6 months and stayed relatively stable through 24 months. Viral pathogens including adenovirus 40/41, astrovirus, norovirus GII, rotavirus, and sapovirus tended to decline in the second year of life (Figure 2). While certain pathogens had a relatively constant burden throughout the year, others demonstrated seasonal patterns, including adenovirus 40/41, *C. jejuni/coli*, *Cryptosporidium* spp, sapovirus, *Vibrio cholerae*, and *Salmonella* spp (Supplementary Figure 2).

### Linear Growth

Anthropometry was available on 179 children at 12 months of age who had continuous diarrheal surveillance and complete covariate data. In regression models without selection, astrovirus AFe was the only pathogen significantly associated with growth (Supplementary Figure 3). With LAZ at 12 months as the outcome, multivariable stepwise regression selected LAZ at enrollment (regression coefficient [RC], 0.36 [95% CI, .18 to .53]), mother’s weight (RC, 0.01 [95% CI, .0001 to .03]), ≥5 people living in the home (RC, –0.36 [95% CI, –.65 to –.08]), and CRP at 18 weeks of age (RC, –0.01 [95% CI, –.02 to –.002]) as significant predictors at the *P* ≤ .05 level. Additionally, astrovirus AFe was inversely associated with LAZ at 12 months of age (RC, –0.63 [95% CI, –1.07 to –.19]).

Anthropometry was available on 180 children at 24 months who had continuous diarrheal surveillance and complete covariate data. These 180 children did not differ from the 70 children excluded from our analysis in baseline characteristics or socioeconomic variables except for food insecurity, which was higher in the included group (98.3% vs 91.4%; Supplementary Table 1). LAZ at enrollment (RC, 0.30 [95% CI, .12 to .48]), mother’s height (RC, 0.01 [95% CI, .0001 to .03]), mother’s weight (RC, 0.02 [95% CI, .003 to .03]), income (RC, 0.01 [95% CI, .002 to .03]), ≥5 people living in the home (RC, –0.48 [95% CI, –.77 to –.19]), additional siblings in the home <5 years of age (RC, –0.42 [95% CI, –.72 to –.01]), use of municipal drinking water (RC, –1.86 [95% CI, –3.55 to –.02]), and sCD14 at 18 weeks (RC, –0.0004 [95% CI, –.001 to –.0002]) were significant at the *P* ≤ .05 level with LAZ at 24 months as the outcome. Norovirus GII AFe was associated with improved LAZ at 24 months (RC, 0.42 [95% CI, .04 to .80]) (Table 1).

**Table 1.** Multivariable Regression With Stepwise Selection to Predict Length-for-Age z Score at 12 and 24 Months

Predictor	LAZ at 12 mo (n = 179)		LAZ at 24 mo (n = 180)	
	Regression Coefficient (95% CI)	*P* Value	Regression Coefficient (95% CI)	*P* Value
Sex	ns	ns	ns	ns
LAZ at enrollment, SD	0.36 (.18–.53)	<.001	0.30 (.12 to .48)	.002
WAZ at enrollment, SD	0.13 (–.06 to .32)	.19	0.13 (–.06 to .32)	.19
Birth during the wet season (Apr–Sep)^a^	-0.23 (–.48 to .01)	.06	–0.22 (–.48 to .04)	.10
Gestational age at birth, wk	ns	ns	ns	ns
Mother’s height, cm	0.01 (–.002 to .02)	.11	.01 (.0001 to .03)	.05
Mother’s weight 4 mo postpartum, kg	0.01 (.0001–.03)	.05	0.02 (.003 to .03)	.02
Mother’s age at child enrollment, y	0.02 (–.008 to .05)	.17	ns	ns
Mother’s age at first pregnancy, y	0.03 (–.01 to .08)	.18	ns	ns
Formal maternal education (none vs any)^a^	ns	ns	ns	ns
Income (per thousand taka^b^)	0.01 (–.0004 to .02)	.06	0.01 (.002 to .03)	.02
≥5 people per household^a^	–0.36 (–.65 to –.08)	.01	–0.48 (–.77 to –.19)	.002
Additional siblings <5 y old in dwelling^a^	–0.27 (–.55 to .01)	.06	–0.42 (–.72 to –.01)	.01
Food deficit as assessed by a family member^a^	ns	ns	ns	ns
Duration of exclusive breastfeeding, d	ns	ns	ns	ns
Flush toilet^a^	ns	ns	0.23 (–.05 to .52)	.11
Concrete floor in dwelling^a^	ns	ns	ns	ns
Kitchen in dwelling	ns	ns	ns	ns
Municipal drinking water^a^	–1.10 (–2.69 to .49)	.17	–1.86 (–3.55 to –.02)	.03
Routine treatment of drinking water (boiling or chlorine vs none)^a^	ns	ns	ns	ns
Open drain outside dwelling^a^	ns	ns	ns	ns
Total days of diarrhea	ns	ns	ns	ns
CRP measurement at 18 wk, μg/mL	–0.01 (–.02 to –.002)	.02	ns	ns
sCD14 measurement at 18 wk, ng/mL	ns	ns	–0.0004 (–.001 to –.0002)	.001
MPO measurement at 22 wk, ng/mL	ns	ns	ns	ns
Reg1β measurement at 22 wk, μg/mL	ns	ns	ns	ns
Adenovirus 40/41^c^	–0.13 (–.46 to .21)	.47	–0.13 (–.38 to .12)	.30
*Aeromonas* ^c^	–1.06 (–2.93 to .80)	.27	–0.27 (–1.45 to .90)	.65
Astrovirus^c^	–0.63 (–1.07 to –.19)	.006	–0.23 (–.58 to .13)	.21
*Campylobacter jejuni/coli* ^c^	–0.28 (–.03 to .58)	.08	–0.03 (–.23 to .17)	.76
*Cryptosporidium* ^c^	0.37 (–.87 to .14)	.16	–0.11 (–.44 to .22)	.50
Norovirus GII^c^	0.31 (–.13 to .75)	.17	0.42 (.04–.80)	.03
Rotavirus^c^	–0.01 (–.24 to .21)	.93	–0.06 (–.26 to .13)	.52
*Salmonella* ^c^	–0.73 (–1.97 to .52)	.26	0.37 (–.83 to 1.57)	.54
Sapovirus^c^	–0.23 (–.63 to .17)	.25	–0.03 (–.35 to .29)	.85
*Shigella*/EIEC^c^	0.05 (–.15 to .25)	.60	–0.02 (–.14 to .11)	.81
*Vibirio cholerae* ^c^	0.21 (–.22 to .64)	.34	0.06 (–.30 to .42)	.74
Typical EPEC^c^	–0.33 (–.97 to .30)	.31	–0.08 (–.69 to .52)	.79
ST-ETEC^c^	0.09 (–.18 to .36)	.51	0.12 (–.12 to .36)	.33

Abbreviations: CI, confidence interval; CRP, C-reactive protein; EIEC, enteroinvasive *Escherichia coli*; EPEC, enteropathogenic *Escherichia coli*; LAZ, length-for-age *z* score; MPO, myeloperoxidase; ns, not significant (variable was dropped by stepwise regression algorithm); Reg 1β, regenerating family member 1β; sCD14, soluble CD14; SD, standard deviation; ST-ETEC, heat-stable toxin–producing enterotoxigenic *Escherichia coli*; WAZ, weight-for-age *z* score.

^a^Dichotomous variable.

^b^One US dollar = 76.69–79.14 Bangladeshi taka over the study duration.

^c^Attributable fraction of diarrhea due to that pathogen over 12 or 24 months.

Regression coefficients for pathogens should be interpreted as change in outcome per 1 attributable episode of diarrhea due to that pathogen.

### Neurodevelopment

One hundred sixty-two children had complete data sets including Bayley-III testing at 24 months of age. The mean composite scores (with standard deviations) were 88.63 (±5.80) for cognitive, 93.50 (±7.44) for language, and 90.65 (±6.12) for motor. With cognitive score as the outcome, our model selected LAZ at enrollment (RC, 1.33 [95% CI, .46 to 2.23]), income (RC, 0.09 [95% CI, .03 to .10]), additional siblings in the home <5 years old (RC, –3.11 [95% CI, –5.03 to –1.19]), presence of a kitchen in the home (RC, –1.83 [95% CI, –3.57 to –.08]), norovirus AFe (RC, 2.46 [95% CI, .05 to 4.87]), sapovirus AFe (RC, –2.64 [95% CI, –4.80 to –.48]), and typical EPEC AFe (RC, –4.14 [95% CI, –8.02 to –.27]) as significant predictors. For language, the model selected female sex (RC, 3.36 [95% CI, 1.22 to 5.49]), mother’s weight (RC, 0.20 [95% CI, .10 to .30]), ≥5 people living in the home (RC, –2.37 [95% CI, –4.67 to –.07]), additional siblings in the home <5 years old (RC, –3.04 [95% CI, –5.39 to –.68]), and treatment of drinking water (RC, 3.30 [95% CI, 1.02 to 5.58]) as significant. Motor score was associated with LAZ at enrollment (RC, 1.08 [95% CI, .04 to 2.12]) and mother’s weight (RC, 0.13 [95% CI, .03 to .22]). No pathogen AFes were associated with language or motor scores (Table 2).

**Table 2.** Multivariable Regression With Stepwise Selection to Predict Bayley-III Scores at 24 Months (n = 162)

Predictor	Cognitive		Language		Motor	
	Regression Coefficient (95% CI)	*P* Value	Regression Coefficient (95% CI)	*P* Value	Regression Coefficient (95% CI)	*P* Value
Female sex^a^	ns	ns	3.36 (1.22–5.49)	.002	ns	ns
LAZ at enrollment (SD)	1.33 (.46 to 2.23)	.005	0.89 (–.10 to 1.98)	.11	1.08 (.04 to 2.12)	.04
WAZ at enrollment (SD)	ns	ns	ns	ns	ns	ns
Birth during the wet season (Apr–Sep)^a^	ns	ns	–1.99 (–4.03 to .04)	.06	ns	ns
Gestational age at birth, wk	ns	ns	ns	ns	ns	ns
Mother’s height, cm	–0.07 (–.16 to .03)	.17	ns	ns	ns	ns
Mother’s weight 4 mo postpartum, kg	0.07 (–.02 to .16)	.12	0.20 (.10 to.30)	<.001	0.13 (.03 to .22)	.01
Mother’s age at child enrollment, y	ns	ns	ns	ns	ns	ns
Mother’s age at first pregnancy, y	ns	ns	ns	ns	ns	ns
Formal maternal education (none vs any)^a^	ns	ns	ns	ns	ns	ns
Income (per thousand taka^b^)	0.09 (.03–.10)	.008	0.08 (–.004 to .17)	.06	ns	ns
≥5 people in household^a^	ns	ns	–2.37 (–4.67 to –.07)	.05	ns	ns
Additional siblings <5 y old in dwelling^a^	–3.11 (–5.03 to –1.19)	.002	–3.04 (–5.39 to –.68)	.01	ns	ns
Food deficit as assessed by family member^a^	ns	ns	ns	ns	ns	ns
Duration of exclusive breastfeeding, d	ns	ns	ns	ns	ns	ns
Flush toilet^a^	ns	ns	ns	ns	1.83 (–.20 to 3.86)	.08
Concrete floor in dwelling^a^	ns	ns	ns	ns	ns	ns
Kitchen in dwelling^a^	–1.83 (–3.57 to –.08)	.04	ns	ns	–1.83 (–3.78 to .12)	.07
Municipal drinking water^a^	ns	ns	ns	ns	ns	ns
Routine treatment of drinking water (boiling or chlorine)^a^	1.57 (–.35 to 3.50)	.11	3.30 (1.02 to 5.58)	.005	ns	ns
Total days of diarrhea	ns	ns	0.06 (–.02 to .04)	.12	0.06 (–.007 to .13)	.08
CRP measurement at 18 weeks, μg/mL	ns	ns	ns	ns	ns	ns
sCD14 measurement at 18 wk, ng/mL	ns	ns	ns	ns	ns	ns
MPO measurement at 22 wk, ng/mL	–0.0001 (–.0002 to .00002)	.13	ns	ns	ns	ns
Reg1β measurement at 22 wk, μg/mL	0.009 (–.004 to .02)	.18	ns	ns	ns	ns
Adenovirus 40/41^c^	–0.84 (–2.41 to .73)	.30	–0.98 (–2.90 to .94)	.32	–0.98 (–2.80 to .84)	.29
*Aeromonas* ^c^	2.05 (–5.58 to 9.68)	.60	0.81 (–8.36 to 9.98)	.86	3.58 (–4.99 to 12.15)	.41
Astrovirus^c^	–0.89 (–3.14 to 1.37)	.44	–0.57 (–3.29 to 2.15)	.68	–1.63 (–4.24 to .97)	.22
*Campylobacter jejuni/coli* ^c^	0.46 (–.86 to 1.78)	.50	–0.24 (–1.89 to 1.40)	.77	–0.79 (–2.32 to .74)	.31
*Cryptosporidium* ^c^	0.37 (–1.89 to 2.63)	.75	1.33 (–1.28 to 3.94)	.32	2.10 (–.35 to 4.55)	.10
Norovirus GII^c^	2.46 (.05 to 4.87)	.05	1.00 (–1.93 to 3.93)	.51	2.48 (–.26 to 5.23)	.08
Rotavirus^c^	0.38 (–.91 to 1.67)	.57	–0.42 (–1.94 to 1.10)	.59	–1.32 (–2.74 to .10)	.07
*Salmonella* ^c^	0.99 (–6.60 to 8.59)	.80	1.63 (–7.67 to 10.93)	.73	–4.83 (–13.63 to 3.96)	.28
Sapovirus^c^	–2.64 (–4.80 to –.48)	.02	–0.36 (–3.05 to 2.32)	.79	–2.41 (–4.92 to .09)	.06
*Shigella*/EIEC^c^	0.21 (–.58 to 1.01)	.60	–0.74 (–1.68 to .21)	.13	0.57 (–.33 to 1.48)	.22
*Vibrio cholerae* ^c^	0.42 (–1.90 to 2.73)	.72	0.71 (–2.04 to 3.46)	.62	2.43 (–.20 to 5.06)	.07
Typical EPEC^c^	–4.14 (–8.02 to –.27)	.04	0.64 (–4.01 to 5.28)	.79	–2.71 (–7.04 to 1.61)	.22
ST-ETEC^c^	–0.13 (–1.67 to 1.41)	.87	0.49 (–1.36 to 2.35)	.60	–0.90 (–2.61 to .81)	.31

Abbreviations: CI, confidence interval; CRP, C-reactive protein; EIEC, enteroinvasive *Escherichia coli*; EPEC, enteropathogenic *Escherichia coli*; LAZ, length-for-age *z* score; MPO, myeloperoxidase; ns, not significant (variable was dropped by stepwise regression algorithm); Reg 1β, regenerating family member 1β; sCD14, soluble CD14; SD, standard deviation; ST-ETEC, heat-stable toxin–producing enterotoxigenic *Escherichia coli*; WAZ, weight-for-age *z* score.

^a^Dichotomous variable.

^b^One US dollar = 76.69–79.14 Bangladeshi taka over the study duration.

^c^Attributable fraction of diarrhea due to that pathogen over 24 months.

## Discussion

The most important finding of this study was that predictors representing maternal and prenatal factors comprise the majority of significant predictors of childhood stunting and neurodevelopment, but certain diarrheagenic pathogens were also predictive. This is consistent with previous work demonstrating the importance of maternal and prenatal factors on growth [29].

Our findings did not replicate those of Schnee et al in a similar Bangladeshi cohort that found *C. jejuni/coli*, *Cryptosporidium* spp, and *Shigella* to be associated with decreased linear growth at 12 months and *C. jejuni/coli* and *Cryptosporidium* spp in the first year of life to be associated with decreased growth at 24 months [28]. *Campylobacter* AFe approached significance in year 1 but was clearly not associated with LAZ over 2 years of life, suggesting that the effects of *Campylobacter* seen in the Schnee et al study might diminish throughout the second year of life and/or the first year might be more critical to long-term growth. The discrepancy between studies may also be due to a difference in sample size, as the Schnee et al analysis had 700 children and we had 180. Also, our analysis had an extensive list of covariates, which may have acted as confounders in previous studies. The finding of norovirus GII being associated with improved growth was noted in both the Schnee et al analysis and ours. Asymptomatic cryptosporidiosis is associated with decreased linear growth at 2 years [21], an effect we may have missed due to only analyzing diarrheal stool.

This work is the first to examine the effects of diarrheagenic pathogens on neurodevelopment. Norovirus GII was not only associated with improved linear growth but also improved cognitive development. Unlike many of the enteric pathogens tested, human noroviruses primarily target antigen-presenting cells in the intestine, suggesting a need for functional intestinal immunity to cause disease [30]. Environmental enteric dysfunction (EED) is a subclinical condition common in LMIC children and hallmarked by intestinal inflammation, dysbiosis, and disrupted intestinal immune homeostasis [16, 31, 32]. EED is pervasive in the Bangladeshi community in which this study was conducted and has been associated with stunting and poor neurodevelopmental outcomes [29, 33–35]. While we included 1 early time point measure of EED, it may be that EED later in life was a confounder in our analysis and children with a lesser degree of EED had improved outcomes as well as increased risk of norovirus infection due to less immune dysregulation. It is also possible there were confounders we did not measure. The nature of the negative effect of EPEC and sapovirus on neurodevelopment is unclear, although murine studies suggest the possibility of a pathogen-derived intestinal dysbiosis affecting the gut–brain axis [36].

Our results concerning pathogen burden were relatively consistent with findings from the Etiology, Risk Factors, and Interactions of Enteric Infections and Malnutrition and the Consequences for Child Health and Development (MAL-ED) study, which examined pathogen burden across 8 sites in South America, sub-Saharan Africa, and Asia including Bangladesh [28, 37, 38]. EAEC, ETEC, *Campylobacter*, and typical EPEC were the most commonly detected pathogens with rotavirus, *Campylobacter*, *Shigella*, ST-ETEC, and adenovirus 40/41 accounting for the most diarrheal disease. Increased *Shigella* detection in the second year of life correlates with a significant rise in the burden of *Shigella* diarrhea. There was more *Campylobacter*-associated diarrhea in our cohort than in the MAL-ED Bangladesh cohort [38]. These variations are likely due to differences in circulating outbreaks that occurred during the various study periods and highlight the outbreak-driven nature of diarrheal disease, which complicates both investigation and empiric clinical intervention. This work should add to previous studies validating resource utilization for vaccine development and implementation for the most common enteric pathogens.

Our study had several strengths. We utilized a birth cohort with frequent home visitation to ensure episodes of diarrhea were captured and used a highly sensitive molecular diagnostic to measure a broad array of enteric pathogens. Also, an intensive list of socioeconomic covariates was corrected for, which limited potential confounders. This work also has several important limitations that should be considered when interpreting our results. Our sample size was relatively low compared to other similar assessments and may have been underpowered to detect smaller effect sizes. This may have led to our analysis missing significant contributions of pathogens to growth and neurodevelopment. Furthermore, Bangladeshi children carry an average of 3.3 enteric pathogens in nondiarrheal stool [39]. Subclinical infections, particularly of the enteric protozoa, have previously been associated with linear growth shortfalls [21, 40, 41]. It may be that important relationships between subclinical enteric infection and growth or neurodevelopment were missed since we only analyzed diarrheal specimens. Finally, synergistic effect of co-pathogen carriage was not assessed but may be a significant driver of pathogenicity [42].

This is the first study to examine the association between specific diarrheagenic pathogens and neurodevelopment. We found that norovirus GII was associated with improved cognitive scores as well as growth. Sapovirus and typical EPEC were associated with poorer cognitive scores. These effects are independent from enteric and systemic inflammation as measured. Our findings suggest that understanding the link between norovirus and growth may provide valuable insights into the pathogenesis of growth and neurodevelopmental delay. Furthermore, our results suggest that sapovirus, an understudied pathogen in LMIC children, and typical EPEC may play a role in poor cognitive outcomes, links that need further exploration.

## Supplementary Data

Supplementary materials are available at *Clinical Infectious Diseases* online. Consisting of data provided by the authors to benefit the reader, the posted materials are not copyedited and are the sole responsibility of the authors, so questions or comments should be addressed to the corresponding author.

ciaa1938_suppl_Supplementary_Figure-1

ciaa1938_suppl_Supplementary_Figure-2

ciaa1938_suppl_Supplementary_Figure-3

ciaa1938_suppl_supplementary_Table-1

## Notes

***Acknowledgments*.** The authors acknowledge the families participating in the Crypto Burden Study and the community of Mirpur, Dhaka, Bangladesh, without whom this endeavor would not have been possible. The authors also thank Dr Eric Houpt for his support and guidance.

***Financial support.*** This work was supported by the National Institutes of Health (NIH) (grant numbers R01 AI043596 and R37 AI026649 to W. A. P. and 1K23HD097282 to J. R. D.) and the Bill & Melinda Gates Foundation.

***Potential conflicts of interest.*** J. R. D. reports grants from the NIH, during the conduct of this study. U. N. reports grants from the Bill & Melinda Gates Foundation, during the conduct of this study. W. A. P. reports grants from the NIH and the Bill & Melinda Gates Foundation during the conduct of this work, as well as from TechLab and Syneos, outside of this work. All other authors report no potential conflicts.
