# Supplementary figures and images for: Diarrheal Pathogens Associated With Growth and Neurodevelopment
Source: Clin Infect Dis. 2021 Jan 5;73(3):e683–91. doi: 10.1093/cid/ciaa1938 (PMC8326554; doi:10.1093/cid/ciaa1938)

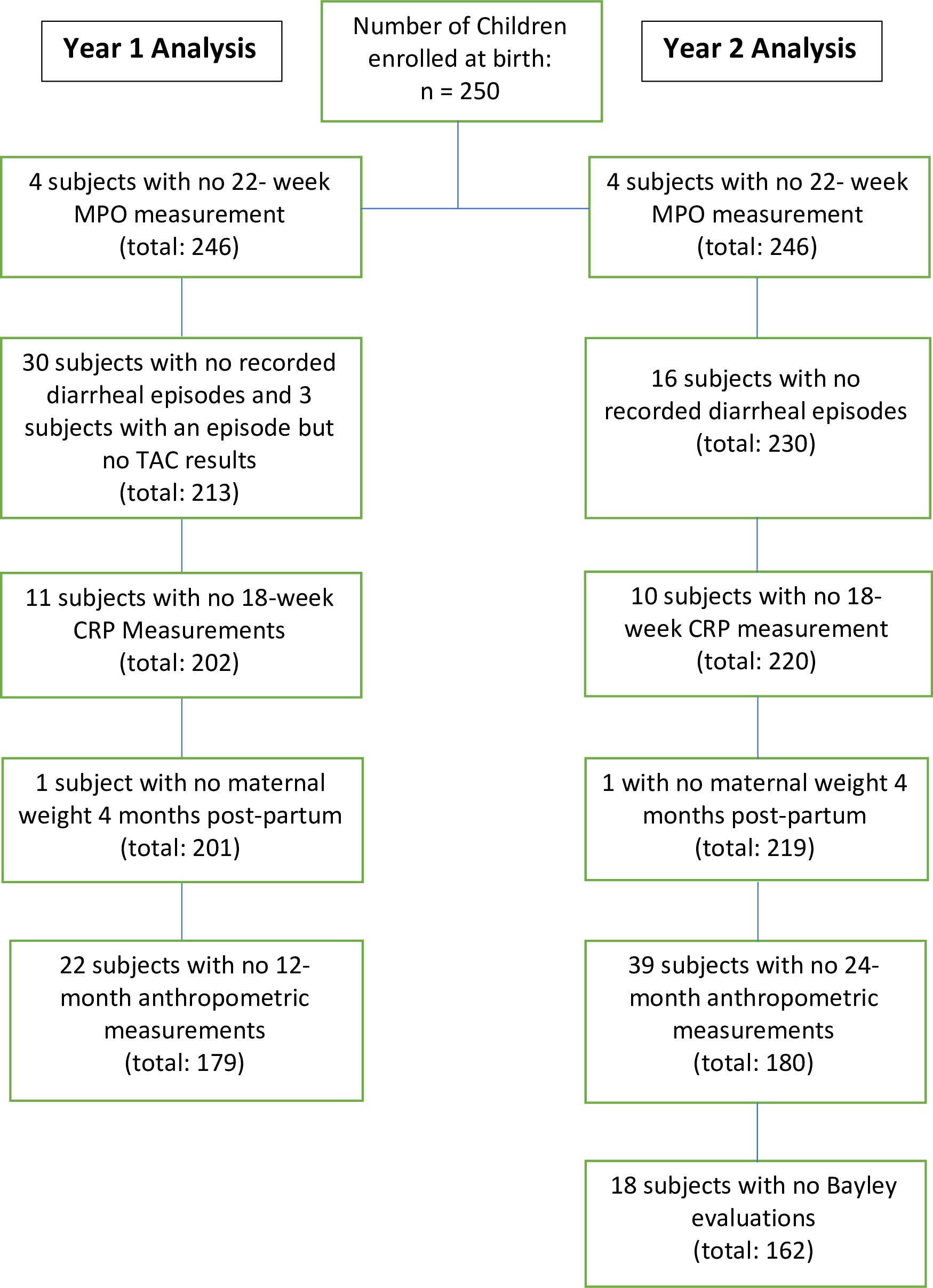

Supplement: ciaa1938_suppl_Supplementary_Figure-1 [file ciaa1938_suppl_supplementary_figure-1.jpeg]

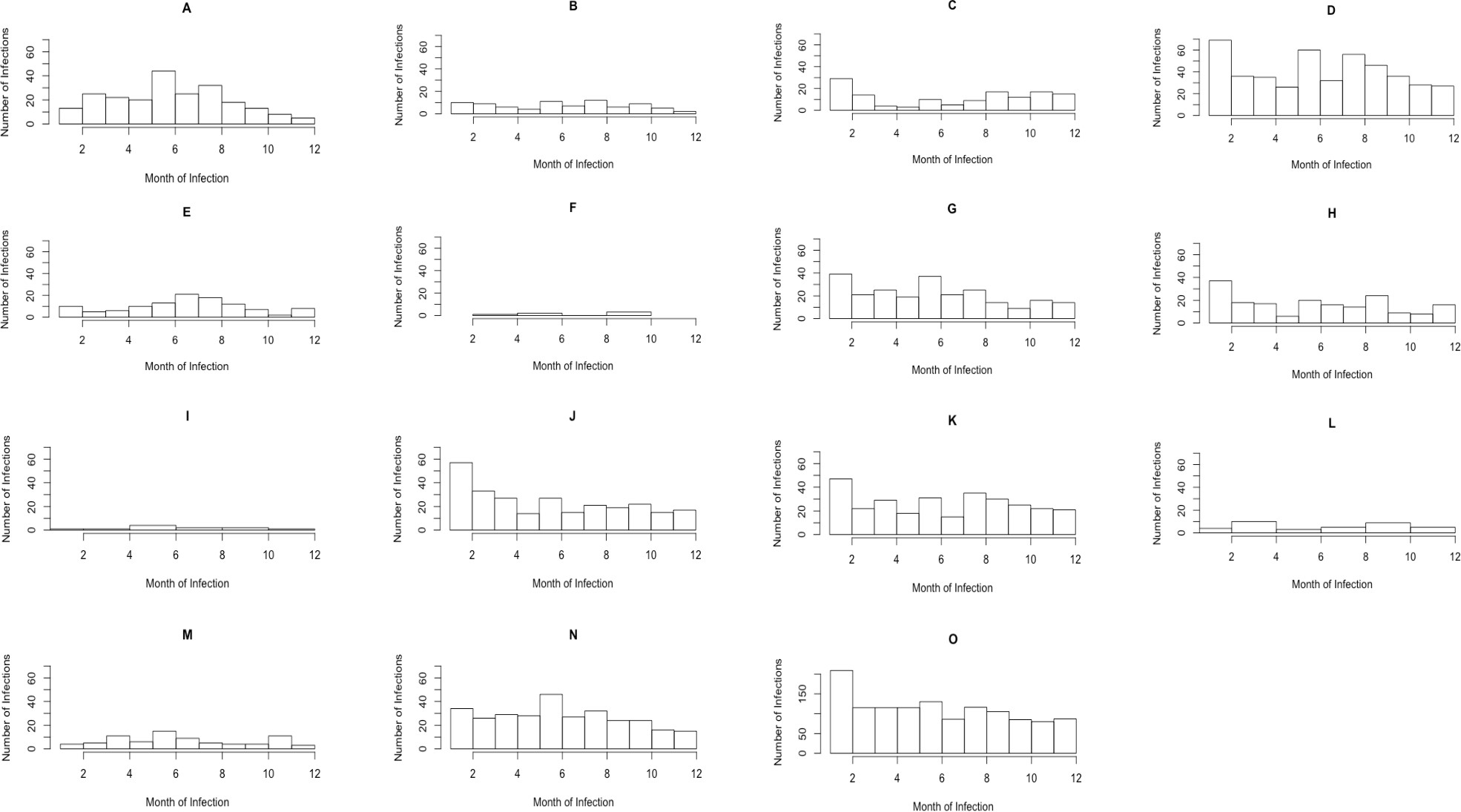

Supplement: ciaa1938_suppl_Supplementary_Figure-2 [file ciaa1938_suppl_supplementary_figure-2.jpeg]

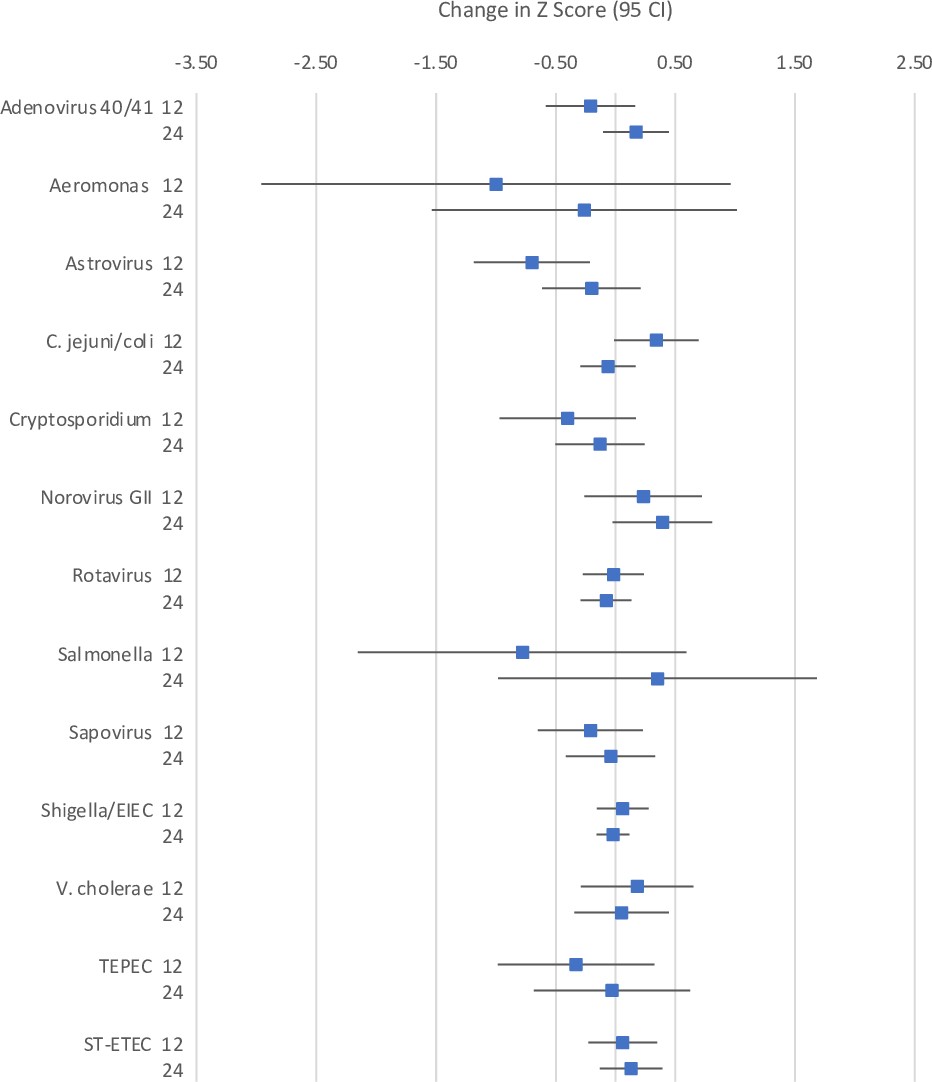

Supplement: ciaa1938_suppl_Supplementary_Figure-3 [file ciaa1938_suppl_supplementary_figure-3.jpeg]
