# Supplementary material for: Diarrheal Pathogens Associated With Growth and Neurodevelopment
Source: Clin Infect Dis. 2021 Jan 5;73(3):e683–91. doi: 10.1093/cid/ciaa1938 (PMC8326554; doi:10.1093/cid/ciaa1938)
Supplement: ciaa1938_suppl_supplementary_Table-1 [file ciaa1938_suppl_supplementary_table-1.docx]

| **Supplementary Table 1**. **Baseline characteristics of those included in our primary analysis of LAZ at 24 months versus those excluded for incomplete data sets.** | | | |
| --- | --- | --- | --- |
| **Variable** | **Included in analysis**  n=180 | **Excluded from analysis**  n=70 | **p value** |
| Female | 99 (55.0%) | 43 (61.4%) | 0.44* |
| LAZ at Enrollment (SD) | -0.88 (+0.9) | -1.15 (+0.9) | 0.07^#^ |
| WAZ at Enrollment (SD) | -1.25 (+0.9) | -1.40 (+0.9) | 0.21^#^ |
| Birth during the wet season (Apr-Sep) | 75 (41.7%) | 32 (44.3%) | 0.66* |
| Gestational age at birth in weeks | 37.9 (+1.7) | 38.0 (+2.0) | 0.83^#^ |
| Mother’s Height (cm) | 149.2 (+9.2) | 149.7 (+5.0) | 1.00^#^ |
| Mother’s Weight 4 months postpartum (kg) | 51.1 (+10.2) | 52.1 (+9.1) | 0.36^#^ |
| Mother’s age at child enrollment (years) | 23.9 (+4.3) | 24.2 (+4.6) | 0.65^#^ |
| Mother’s age at first pregnancy (years) | 19.1 (+2.6) | 19.0 (+2.3) | 0.75^#^ |
| Mother with no formal education | 40 (22.2%) | 12 (17.1%) | 0.48* |
| Income (per thousand taka) | 17.3 (+13.2) | 17.3 (+21.9) | 0.14^#^ |
| >5 people in the home | 72 (40.0%) | 24 (34.3%) | 0.49* |
| No additional siblings under 5 years old in the home | 134 (74.4%) | 51 (72.9%) | 0.92* |
| No food deficit as assessed by family member | 177 (98.3%) | 64 (91.4%) | 0.02* |
| Duration of exclusive breastfeeding (days) | 114.7 (+68.7) | 114.2 (+72.0) | 0.92^#^ |
| No Flush toilet | 113 (62.8%) | 44 (62.9%) | 1.00* |
| No concrete floor in dwelling | 5 (2.8%) | 3 (3.8%) | 0.84* |
| No kitchen in dwelling | 107 (59.4%) | 43 (53.8%) | 0.89* |
| Uses municipal drinking water | 179 (99.4%) | 70 (87.5%) | 1.00* |
| No routine treatment of drinking water | 47 (26.1%) | 18 (22.5%) | 1.00* |
| N.B. Dichotomous variables expressed as count (%); continuous variables expressed as mean (SD)  *Chi-squared analysis  ^#^Wilcoxon (Mann-Whitney U) | | | |

**Supplementary Figure 1.** Children included in analysis of growth and neurodevelopment. Only children with complete data sets were included in the final analysis. 180 children were included for our model of Length-for-age Z score at 2 years and 162 children for Bayley-III Scales of Infant and Toddler Development scores at 2 years of age.

**Supplementary Figure 2.** Seasonality of pathogens as detected using the TaqMan Array Card. Seasonal variation in pathogen detection is shown for Adenovirus 40/41 (Panel A), *Aeromonas* (Panel B), Astrovirus (Panel C), *C. jejuni/coli* (Panel D), *Cryptosporidium spp.* (Panel E), *Isospora* (Panel F), norovirus GII (Panel G), rotavirus (Panel H), *Salmonella* spp. (Panel I), sapovirus (Panel J), *Shigella* (Panel K), *V. cholerae* (Panel L); Typical Enteropathogenic *E. coli* (Panel M), heat-stable toxin producing Enterotoxigenic *E. coli* (ST-ETEC) (Panel N), and for all 14 enteropathogens combined (Panel O).

**Supplementary Figure 3.** Multivariable linear regression model without selection to predict length-for-age Z score (LAZ) at 12 and 24 months of age. Regression coefficients and 95% confidence intervals are plotted. Pathogen attributable fraction (AFes) are corrected for socioeconomic variables collected (as shown in Tables 1 and 2). Our model demonstrated that only Astrovirus was associated with LAZ at 12 months of life (Regression Coefficient -0.70; 95% Confidence Interval -1.20 – -0.21). No pathogen was associated with LAZ at 24 months of life.
